# DNA demethylation marks in chronic lymphocytic leukemia: it is time to let the cat out of the bag

**DOI:** 10.4155/fsoa-2017-0120

**Published:** 2017-11-03

**Authors:** Cristina Bagacean, Mihnea Zdrenghea, Christelle Le Dantec, Adrian Tempescul, Christian Berthou, Yves Renaudineau

**Affiliations:** 1 U1227 B Llymphocytes & Autoimmunity, University of Brest, INSERM, IBSAM, Brest, France; 2 Labex IGO, Networks IC-CGO & REpiCGO from ‘Canceropole Grand Ouest’, Brest, France; 3 Laboratory of Immunology & Immunotherapy, Brest University Medical School Hospital, Brest, France; 4 ‘Iuliu Hatieganu’ University of Medicine & Pharmacy, Cluj-Napoca, Romania; 5 Department of Hematology, Brest University Medical School Hospital, Brest, France

**Keywords:** chronic lymphocytic leukemia, DNA hydroxymethylation, DNA methylation, DNMT, TET

In chronic lymphocytic leukemia (CLL), DNA methylation changes are essential in the pathogenesis of the disease and lead to the identification of potential biomarkers relevant for clinical follow-up and personalized medicine. Accordingly, understanding the mechanisms of controlling DNA methylation/demethylation is critical for future biomarker and therapeutic development in CLL.

The dynamic interplay between DNA methylation and DNA demethylation mechanisms in maintaining normal chromatin structure and gene expression in normal cells and the causes and consequences of an imbalance between the two processes are critical points highly discussed in recent years. The debate was recently reinforced by the description of an active DNA demethylation pathway by which 5-methylcytosine (5-mCyt) is sequentially converted to 5-hydroxymethylcytosine (5-hmCyt) and, in a less efficient fashion, to 5-formylcytosine and 5-carboxylcytosine by TET dioxygenases. This point is critical, as a major limitation that has delayed advancement of our knowledge on this subject was related to a technical limitation based on the impossibility to distinguish 5-hmCyt from 5-mCyt after bisulfite treatment. This limited accurate detection and quantification of the levels of 5-hmCyt at single-base resolution. Indeed, the majority of studies published so far on genome-wide epigenetic mark modifications have used microarray or sequencing techniques based on bisulfite treated DNA. Therefore, the assertion that 5-mCyt, considered as the ‘fifth base’, has a critical role *per se* in the development and the pathogenesis of many diseases, including cancer initiation and progression, must be re-evaluated. The classical view is that 5-mCyt modifications cause silencing of tumor suppressor genes and activation of oncogenes [1]. However, there are several studies that provide grounds for skepticism regarding this assertion, such as the recently published study of Zhang *et al*. that reveals a poor correlation between 5-mCyt distribution and gene expression, while 5-hmCyt displays a ‘bimodal’ pattern of regulation highly correlated with gene expression [2]. Overexpressed genes were associated with high 5-hmCyt in the promoters and gene bodies, while lower relative gene expression was associated with higher 5-hmCyt at the transcription start sites [2].

Even though 5-hmCyt was initially considered to be just an intermediate in the DNA demethylation process rather than an important epigenetic mark *per se* with distinct regulatory functions from 5-mCyt, this second assertion is currently accepted to a greater extent. This hypothesis is sustained on one hand by the stability of 5-hmCyt marks during the cell cycle, even though it takes more time to establish these marks on the newly synthesized DNA strand compared with 5-mCyt and, on the other hand, by the high tissue specificity of global 5-hmCyt levels [3,4]. Moreover, TET1 and TET2 preferential catalytic activity has been described for the 5-mCyt substrate, this activity being reduced 4.9–6.3-fold for 5-hmCyt, and even more for 5-formylcytosine (7.8–12.6-fold) [5]. The substrate preference is due to inter- and intra-molecular hydrogen bonds which confer a more restrained conformation of the hydroxyl and carbonyl groups, compared with the methyl group [5]. In other words, after 5-hmCyt formation, the oxidation activity of TET is reduced, leading to accumulation of a stable 5-hmCyt pattern in the genome with regulatory functions.

The contribution of 5-hmCyt to DNA repair represents another recent subject of interest. 5-hmCyt has been reported as a covalent modification arising at sites of DNA damage, colocalizes with several repair factors (53BP1, Rad51 and γH2AX) and promotes genome stability [6]. Moreover, TET1 and TET3 have been shown to be phosphorylated by two DNA damage response-activated kinases, ATM and ATR, respectively, further stimulating 5-hmCyt production [7,8].

The hematopoietic system provides an ideal model system for studying the epigenetically directed changes during differentiation, since it follows a strict hierarchical pattern which begins with the hematopoietic stem cell (HSC). However, the molecular mechanisms that determine cell fate and lineage commitment are still not well understood. The consequences of disturbed epigenetic patterns in hematopoietic cells have generated a myriad of work which revealed that epigenetic regulators such as the DNMT and TET enzymes, as well as the patterns of 5-mCyt and 5-hmCyt, have a key role in the hematopoietic system and its hierarchical cascade of differentiation. Accordingly, the recent study of Tekpli *et al*. offers an integrative view on the variations of 5-hmCyt during differentiation of the hematopoietic CD34^+^ cell progenitors into either lymphoid or myeloid lineages [9]. The study showed a selective occurrence of 5-hmCyt in key genes linked to the differentiation in CD34^+^ cells and a decrease of its density in gene bodies during differentiation [9]. This clearly demonstrates that 5-hmCyt marks key genes required for lineage specification and mature blood cell function. Studies on TET knock-out (KO) mice further support a critical role for 5-hmCyt in myeloid and lymphoid differentiation, and in the emergence of leukemia. First, *Tet1* deletion in mice promotes an aberrant expression of transcriptional programs involved in B-cell lineage specification at early stage, altered chromosome maintenance, DNA repair and the development of B-cell lymphoma [10]. An interesting finding from the same study was that loss of *Tet1* acted together with the overexpression of the proto-oncoprotein BCL-2, a protein consistently overexpressed in CLL, to drive B-cell lymphocytosis [11]. *TET1* deficient HSCs showed global loss of 5-hmCyt and gain in 5-mCyt across all chromosomes, with greater losses in 5-hmCyt occurring in the body of genes, 5-mCyt gain at promoters and increased self-renewal potential [10]. Moreover, ectopic expression of *TET1* in lymphoma cell lines inhibits CpG methylation of tumor suppressor gene promoters and reactivates their expression, supporting its role as a tumor suppressor in lymphoproliferative diseases [12]. Second, loss of *Tet2*, on the other hand, induces a myeloid bias of the HSC and chronic myelomonocytic leukemia in *Tet2* KO mice [13]. However, it has also been shown that loss of *Tet2* associated either with *Dnmt3a* or *Tet1* in double KO mice can result in lymphoid malignancies with a phenotype similar to CLL B cells [2,14]. In humans, our previous work sustains an important concomitant impact of *TET1*, *TET2* and *DNMT3A* aberrant expression in CLL patients with aggressive diseases, as they are the epigenetic regulators with the lowest expression in CLL patients with a global deficit in cytosine derivatives including 5-mCyt and 5-hmCyt and the worst prognosis [15,16]. Moreover, in CLL patients, higher levels of *DNMT3A* and *TET1* were associated with longer treatment-free survival, thus supporting their function as tumor suppressors. Altogether, the data mentioned so far show the critical and complementary role of 5-mCyt and 5-hmCyt marks and their enzymatic regulator function in normal hematopoiesis, lineage skewing and malignant transformation in CLL and, finally, in CLL disease progression.

The impact of epigenetic disturbances on HSC differentiation clearly demonstrated by mouse models, together with increasing evidence that the first oncogenic event in CLL occurs in HSC, as the early driver genetic anomalies have been traced up at this early stage of differentiation, lead us to the hypothesis that the aberrant DNA methylation and hydroxymethylation patterns may also intervene at this stage [17–19]. Epigenetic modifications could further bias HSC commitment to the lymphoid lineage, promote proliferation and epigenetically program CLL cells to a specific state of differentiation. Indeed, CLL B cells from peripheral blood present a gene expression profile similar to memory B cells and an immunophenotype consistent with an activated and antigen experienced B cell [20,21]. However, so far, no data are available on the epigenetic profile of HSC in CLL.

In light of the data generated in the last 2 years, it has become clear that specific genome-wide 5-hmCyt datasets are necessary in both peripheral blood CLL B cells and CLL CD34^+^ HSCs, compared with their normal counterparts, but also for sequential samples of peripheral CLL B cells during stable and progressive disease. Various applications of such studies can be highlighted. First, the 5-hmCyt pattern can be considered as a ‘barcode’ that could assist in identifying and classifying CLL patients in prognostic groups at an early stage of the disease [22]. Another potential use would be for stratification of patients into optimal downstream treatment regimens. A study of the 5-hmCyt marked signaling pathways before and after treatment would allow us to delineate the response of CLL patients to certain drugs. Unlike genetic mutations, epigenetic modifications are potentially reversible. Personalized cancer treatment targets, identified through 5-hmCyt genome-wide studies, can involve certain aberrantly dehydroxymethylated and downregulated tumor suppressor genes but also hydroxymethylated and overexpressed oncogenes. Although TET enzymes seem to have an overall protective function in CLL, aberrant hydroxymethylation of oncogenes is possible. Therefore, epigenetic treatments should be specifically tailored for different targets. Recent technological advances would allow a researcher to manipulate the epigenetic states of various loci in order to identify these novel cancer therapies.
